# Increased Neural Reward Responsivity in Adolescents with ASD after Social Skills Intervention

**DOI:** 10.3390/brainsci10060402

**Published:** 2020-06-24

**Authors:** Elizabeth Baker, Elina Veytsman, Ann Marie Martin, Jan Blacher, Katherine K. M. Stavropoulos

**Affiliations:** Graduate School of Education, UC Riverside, Riverside, CA 92521, USA; ebake001@ucr.edu (E.B.); eveyt001@ucr.edu (E.V.); amart135@ucr.edu (A.M.M.); Jan.Blacher@ucr.edu (J.B.)

**Keywords:** autism spectrum disorder, EEG, ERP, reward response, RewP, sensitization, social skills intervention, PEERS^®^

## Abstract

The reward system has been implicated as a potential neural mechanism underlying social-communication deficits in individuals with autism spectrum disorder (ASD). However, it remains unclear whether the neural reward system in ASD is sensitive to behavioral interventions. The current study measured the reward positivity (RewP) in response to social and nonsocial stimuli in seven adolescents with ASD before and after participation in the Program for the Education and Enrichment of Relational Skills (PEERS^®^) intervention. This study also included seven neurotypical adolescents who were tested at two time points but did not receive intervention. We examined the RewP across the course of a task by comparing brain activity during the first versus second half of trials to understand patterns of responsivity over time. Improvements in social skills and decreased social-communication impairments for teens with ASD were observed after PEERS^®^. Event-related potential (ERP) results suggested increased reward sensitivity during the first half of trials in the ASD group after intervention. Adolescents with ASD who exhibited less reward-related brain activity before intervention demonstrated the greatest behavioral benefits from the intervention. These findings have implications for how neuroscience can be used as an objective outcome measure before and after intervention in ASD.

## 1. Increased Neural Reward Responsivity in Adolescents with ASD after Social Skills Intervention

The cognitive process of habituation can be conceptualized in a variety of ways, but is generally considered a decreased response to stimuli after repeated exposure [1]. Individuals with autism spectrum disorder (ASD), defined by social communication deficits and the presence of restricted interests and repetitive behaviors [2], display altered rates of habituation. Specifically, individuals with ASD do not habituate to social information at the same rate as neurotypical controls, as evidenced through amygdala activation to faces over time [3,4,5,6,7]. In individuals with ASD, repeated presentation of social information elicits activation rates similar to that of novel stimuli for neurotypical subjects [8]. In neurotypical individuals, habituation tends to occur at a lower rate for stimuli that are more salient, intense, or stimulating [1,9]. Salient information may cause sensitization to stimuli, such that heightened responses can be observed over time [1,10]. One explanation for slowed habituation rates in response to faces is that individuals with ASD find processing social information more challenging than their neurotypical peers and thus must employ more cognitive resources. Alternatively, lack of habituation could reflect sensitization in this population.

Beyond reflecting the allocation of cognitive resources, habituation is also an indicator of learning. Reinforcement learning is facilitated by the goal of maximizing rewards and satisfying desired outcomes. The reward system has been discussed at length in relation to the core symptoms of ASD. According to the social motivation hypothesis, individuals with ASD experience social interactions as less rewarding than their neurotypical peers, which may lead to reduced social initiation during critical periods of social development [11]. Investigations utilizing electroencephalography (EEG) to measure reward-specific event-related potentials (ERPs) suggest that children with ASD tend to find nonsocial stimuli more salient than social stimuli, and that children with ASD have less reward-related brain activity than that of their neurotypical peers in response to faces [12]. Thus, it is not that the reward system in ASD populations is under-active in response to all stimulus types, but that it is selectively functioning for some categories and not others [13]. However, the literature is mixed on whether the reward system is globally hypoactive in individuals with ASD [14,15]. If the reward system is selectively functioning in ASD, this system might be malleable, and behavioral intervention strategies that focus on social reinforcement might increase brain activity in response to social stimuli in this population. This hypothesis is supported by previous literature demonstrating neural changes in participants with ASD from pre- to post-intervention [16,17,18,19,20,21,22].

Social skills interventions for individuals with ASD often implement strategies of reinforcement learning, including applied behavior analysis and social skills training [23,24,25]. The goal of many interventions is to provide training for independent skill acquisition, ranging from a reduction in maladaptive behavior to increasing social engagement at school. Considerations of habituation or sensitization before and after such interventions are pertinent to not only the effectiveness of intervention but also the interpretation of outcomes.

Understanding how reward-related brain activity changes across the course of a task for individuals with and without ASD can increase our understanding of whether habituation or sensitization occurs at a similar rate across populations, and whether such activity is affected by participation in a social skills intervention. One method for measuring change in brain activity across a task is analyzing brain activity during the first and second halves of a task separately. In the current study, we sought to understand processes of habituation and sensitization to social stimuli among adolescents with ASD by examining patterns of reward-related neural responses to social versus nonsocial stimuli across a task (e.g., activity in the first versus second half of a task), before and after participation in a social skills intervention. The ERP task utilized was a reward-based guessing game in which participants were presented with rewards accompanied by incidental face or nonface stimuli.

## 2. Methods

### 2.1. Participants

Participants included seven adolescents with ASD, and seven age- and gender-matched neurotypical (TD) adolescents. Detailed information about participant demographics can be found in Table 1. No significant differences in age or IQ were observed between groups (*p’s* > 0.70).

For both the ASD and TD groups, exclusionary criteria included a history of seizures/epilepsy, a history of brain injury or disease, or a diagnosis of intellectual disability. For the TD group, immediate family history of ASD or developmental disabilities, or any psychiatric diagnosis for the adolescent was exclusionary. For the ASD group, a diagnosis of ASD was required, though commonly co-occurring disorders were not exclusionary (e.g., ADHD). For the ASD group, history of serious psychiatric illness (e.g., schizophrenia, bipolar disorders) or a recent (within 6 months) psychiatric hospitalization were exclusionary.

The study took place in inland Southern California with a large Latinx population [26]. Participant families were recruited via flyers posted online and via local community organizations. Those who expressed interest were contacted for an initial phone screen. At the initial intake appointment, informed consent and assent (from adolescents) were obtained.

### 2.2. Behavioral Intervention (Program for the Education and Enrichment of Relational Skills, (PEERS^®^))

PEERS^®^ [25,27,28,29] is a manualized intervention designed to help adolescents make and keep friends (see [30] for intervention details). PEERS^®^ consists of 16 weekly 1.5 h group sessions with concurrent but separate adolescent and parent groups. Parents learn how to support their adolescents in practicing and maintaining skills outside of the group. All groups were run by PEERS^®^ certified providers.

## 3. Measures

Cognitive abilities were assessed using the 2-subtest Wechsler Abbreviated Scales of Intelligence [31] (WASI-II); an IQ under 70 was exclusionary for both groups. For adolescents with ASD, diagnosis was confirmed using the Autism Diagnostic Observation Schedule, Second Edition [32] (ADOS-2), and motivation to learn how to make and keep friends was assessed using the Mental Status Checklist [25]. Trained study staff performed these assessments. As these measures were used to confirm eligibility, they were only completed prior to the intervention.

### 3.1. Questionnaires

Data reported here are part of a larger-scale study. Caregivers completed the Social Responsiveness Scale, Second Edition [33] (SRS-2) and the Social Skills Improvement System [34] (SSIS) both before the intervention began (Time 1), and immediately after intervention completion (Time 2). Times 1 and 2 were approximately 4 months apart. Neurotypical adolescents (TD participants) did not receive PEERS^®^, but had lab visits at Times 1 and 2, where each visit was four months apart. In addition, all adolescents completed the Test of Adolescent Social Skills Knowledge, Revised [27] (TASSK-R) at both Time 1 and Time 2, which measures acquisition of the concepts taught in PEERS^®^.

### 3.2. Electrophysiology Stimuli and Task

The stimuli and task are described in detail in previously published manuscripts [12,35,36]. Briefly, the task was a guessing game in which participants saw a left and right visual stimulus (question marks), and were asked to indicate their guess via button press whether the left or right stimulus was “correct.” After this choice, the left and right question marks were replaced with an arrow in the middle pointing towards whichever question mark the participant chose. This was done to reinforce the idea that participants had control over the task and their responses were being recorded.

In previously published manuscripts utilizing this task, participants were told that the reward for each correct answer was a small snack; here, the food reward was an Oreo cookie, or if preferred, fruit snacks or goldfish crackers. Participants were told that if they guessed correctly, they would see a ring of intact Oreo cookies, and the cookies would be crossed out for incorrect answers. There were two blocked feedback conditions: Social versus nonsocial. Importantly, in both the social and nonsocial feedback trials, the face/arrow information was incidental (e.g., the face/arrow image was not part of the overt task). Thus, differences in brain activity between social and nonsocial conditions were not due to differences in tangible rewards or differences in task structure. Incidental stimuli in the social condition were faces obtained from the NimStim database [37] that were smiling for “correct” answers and frowning for “incorrect” answers. Incidental stimuli in the nonsocial condition were composed of scrambled face elements from the social condition formed into an arrow that pointed upwards for “correct” answers and downwards for “incorrect” answers. The order of social versus nonsocial blocks was counterbalanced between participants.

A computer program predetermined correct versus incorrect answers in a pseudorandom order, such that children got 50% “correct” and 50% “incorrect,” with no more than three of the same answer-type in a row. The two feedback conditions (face/“social” trials and arrow/“nonsocial” trials) were tested in separate blocks, each composed of 50 trials.

### 3.3. EEG Recording

Participants wore a standard, fitted cap (Brain Products ActiCap) with 32 silver/silver-chloride (Ag/AgCl) electrodes placed in accordance with the extended international 10–20 system. Continuous EEG was recorded using a Brain Vision Recorder with a reference electrode at Cz, and re-referenced offline to the average activity at left and right mastoids. Electrode resistance was kept under 50 kOhms. Continuous EEG was amplified with a directly coupled high pass filter (DC), and notch filter (60 Hz). The signal was digitized at a rate of 500 samples per second. Eye movement artifacts and blinks were monitored via horizontal electrooculogram (EOG) placed at the outer canthi of each eye and vertical EOG placed above and below the left eye. Trials were time locked to the onset of the feedback stimulus. To measure reward processing, the baseline period was −100–0 ms, and the data were epoched from −100 to 800 ms. Trials with no behavioral response, or containing electrophysiological artifacts, were excluded.

Artifacts were removed via a four-step process. Data were visually inspected for drift exceeding ±200 mV in all electrodes, high frequency noise visible in all electrodes larger than 100 mV, and flatlined data. Following inspection, data were epoched and eyeblink artifacts were identified using independent component analysis (ICA). Individual components were inspected alongside epoched data, and blink components were removed. To remove additional artifacts, we utilized a moving window peak-to-peak procedure in ERPlab [38], with a 200 ms moving window, a 100 ms window step, and a 150 mV voltage threshold.

For both conditions (face, arrow) and both feedback types (correct, incorrect), mean brain activity was calculated between 275 and 425 ms after feedback onset. The reward positivity (RewP) was defined as a difference wave, wherein brain activity in response to “incorrect” feedback was subtracted from brain activity in response to “correct” feedback. For statistical analysis, mean amplitude of the RewP between 275 and 425 ms was utilized. To compare reward-related brain activity during the first half and second half of trials, the first half and last half of all accepted trials (e.g., trials that were not removed through any of the processes mentioned above) were extracted for each of the two conditions (e.g., faces, arrows). Comparing brain activity during the first and second halves of trials allowed us to better understand patterns of reward-related brain activity throughout the task. To be included in statistical analysis, participants had to have a minimum of 6 trials in each half of each condition.

## 4. Results

All analyses were conducted using SPSS (version 26, Armonk, NY, USA). Prior to analysis, Pearson correlations between ERP amplitude, age, and IQ were conducted. No significant relationships were observed (*p’*s > 0.421).

### 4.1. ERP Results

An independent samples t-test was conducted to ensure no significant differences in the number of acceptable trials were present between groups (all *p’*s > 0.638).

**Figure 1:**
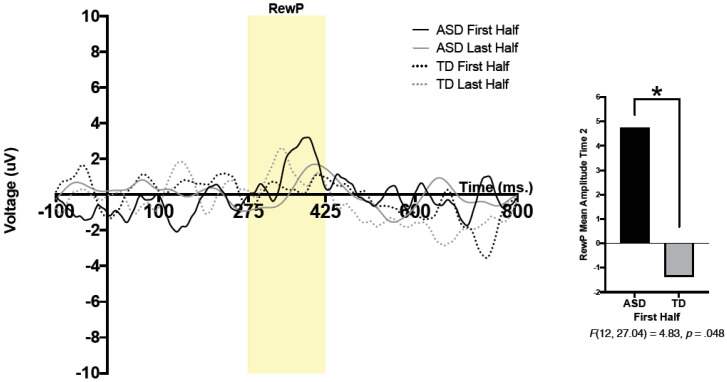
Grand average waveforms during the first and second halves of trials in participants with and without ASD at Time 2 (post-intervention). Significant differences were observed between the ASD and TD groups during the first half of trials at Time 2 (post-intervention). Note that for the purposes of this figure, the ERP was filtered using a 25 Hz low-pass filter. * *p* < 0.05.

A 2 (group) × 2 (condition) × 2 (time) × 2 (half) repeated measure analysis of variance (ANOVA) was run. Condition (social, nonsocial), time (pre-intervention, Time 1; post-intervention, Time 2), and half (RewP amplitude during the first and second halves of the task) were within-subjects variables, and group (TD, ASD) was used as a between-subjects variable. A significant 3-way interaction was found between time, half, and group; *F*(12, 20.76) = 5.20, *p* = 0.042, *η_p_*^2^ = 0.30. Pairwise comparisons revealed a significant effect of group, such that the ASD group had significantly larger RewP amplitude compared to that of the TD group in the first half of trials at Time 2; *F*(12, 27.04) = 4.83, *p* = 0.048. Thus, regardless of condition, the ASD group had larger reward-related brain activity in the first half of presented trials at Time 2 (post-intervention) compared to that of the TD group. No other significant main effects or interactions were observed. See Figure 1 for grand average waveforms at Time 2.

### 4.2. Behavioral Results

To understand how behavioral measures changed over time for each group, 2 (group) × 2 (time) repeated measure ANOVAs were conducted on measures of autism symptoms (SRS-2), social skills (SSIS social skills subscale), and PEERS^®^-specific knowledge (TASSK-R).

For the SRS-2, a main effect of group was observed, *F*(1,12) = 9.51, *p* = 0.009, *η_p_*^2^ = 0.96, such that the TD group had significantly lower SRS-2 scores than those of the ASD group. Lower SRS-2 scores indicate less severe social impairments. An interaction between group and time approached significance, *F*(1, 12) = 4.56, *p* = 0.054. Post-hoc follow-up tests using Bonferroni corrections revealed a significant difference between groups on the SRS-2 at Time 1 (pre-intervention), such that the TD group had lower scores than those of the ASD group (*p* = 0.001). The difference between the two groups was no longer significant at Time 2 (post-intervention). Pairwise comparisons revealed a trend-level effect of time for the ASD group, such that SRS-2 scores decreased from pre- to post- intervention (*p* = 0.07), whereas no effect of time was observed for the TD group.

For the SSIS social skills subscale, an interaction between group and time approached significance, *F*(1,12) = 4.20, *p* = 0.063. Post-hoc follow-up tests using Bonferroni corrections revealed a significant effect of time for the ASD group, such that SSIS social skills subscale scores increased from pre- to post- intervention (*p* = 0.035), whereas no effect of time was observed for the TD group. Higher scores on the SSIS social skills subscale indicate better social skills. Pairwise comparisons also revealed a trend-level difference between groups on the SSIS social skills subscale at Time 1 (pre-intervention) such that the TD group had higher scores than those of the ASD group (*p* = 0.071), whereas the difference between groups was not significant at Time 2 (post-intervention).

For the TASSK-R, a main effect of group was observed, *F*(1,12) = 5.4, *p* = 0.038, *η_p_*^2^ = 0.31, such that adolescents with ASD had higher scores on the TASSK-R compared to neurotypical teens. Higher scores on the TASSK-R indicate more understanding of PEERS^®^-specific skills. A significant effect of time was observed, *F*(1,12) = 45.82, *p* < 0.001 *η_p_*^2^ = 0.79, such that TASSK-R scores increased from Time 1 (pre-intervention) to Time 2 (post-intervention). A significant interaction between time and group was observed, *F*(1,12) = 25.78, *p* < 0.001, *η_p_*^2^ = 0.68. Post-hoc follow-up tests using Bonferroni corrections revealed a significant effect of time for the ASD group, such that scores on the TASSK-R increased from pre- to post-intervention (*p* < 0.001). No effect of time was observed for the TD group. Pairwise comparisons also revealed a significant difference between groups on the TASSK-R at Time 2 (post-intervention), such that the ASD group had higher scores on the TASSK-R compared to those of the TD group (*p* = 0.001), whereas the difference between groups was not significant at Time 1 (pre-intervention). Please refer to Table 2 for behavioral measures at each timepoint.

### 4.3. Brain and Behavior Correlations

**Figure 2:**
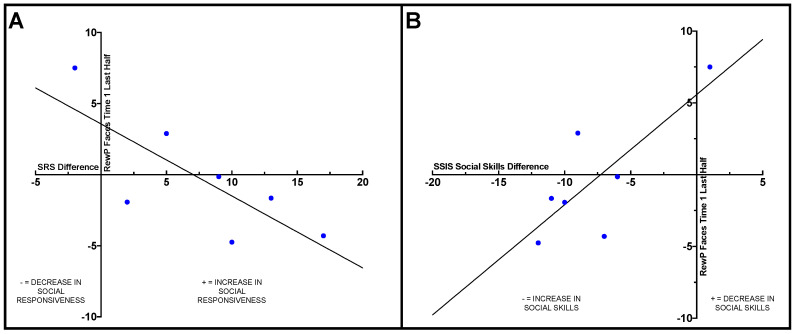
(**A**) Correlation between SRS-2 difference score before and after intervention in the ASD group and reward positivity (RewP) mean amplitude in the last half of the social condition at Time 1 (*r* = −0.77, *p* = 0.04). (**B**) Correlation between SSIS social skills difference score before and after intervention in the ASD group and RewP mean amplitude in the last half of the social condition at Time 1 (*r* = 0.78, *p* = 0.04).

Within the ASD group, Pearson correlations were conducted to examine how change on the behavioral measures from pre- to post-intervention related to ERP results. Difference scores were calculated for the SRS-2, SSIS social skills subscale, and TASSK-R by subtracting post-intervention scores from pre-intervention scores. A significant negative correlation was observed between the SRS-2 difference score and RewP amplitude in the last half of the social condition at Time 1 (*r* = −0.77, *p* = 0.044), such that participants with ASD who had less reward-related brain activity in response to social stimuli at Time 1 (pre-intervention) displayed larger improvements on the SRS-2 compared to individuals with more robust social reward-related brain activity at Time 1. See Figure 2A.

A positive correlation was observed between RewP amplitude in the last half of the social condition at Time 1 (pre-intervention) and SSIS social skills subscale difference score (*r* = 0.78, *p* = 0.038), such that adolescents with ASD who displayed less social reward-related brain activity during the last half of trials in the social condition at Time 1 exhibited greater improvements in social skills from pre- to post-intervention compared to those who displayed more robust reward-related brain activity prior to intervention. See Figure 2B.

Finally, a negative correlation was found between the TASSK-R difference score and RewP amplitude in the last half of the social condition at Time 2 (post intervention) (*r* = −0.79, *p* = 0.035), such that participants with ASD who demonstrated larger increases in their knowledge of intervention-specific knowledge displayed larger social reward-related brain activity in response during the second half of trials compared to participants who had smaller increases in intervention-specific knowledge from pre- to post-intervention.

No significant correlations were observed between behavioral measures and reward-related brain activity in the nonsocial (arrow) condition.

## 5. Discussion

This study investigated the effect of the PEERS^®^ social skills intervention on both neural correlates of reward processing and social behaviors in adolescents with ASD. Specifically, we sought to understand how reward-related brain activity changed throughout the course of a task by comparing brain activity during the first and second halves of trials.

Prior to the start of the intervention, patterns of reward-related brain activity did not differ between participants with ASD and their neurotypical peers. However, after intervention, participants with ASD were more sensitive or responsive to all reward types (both social and nonsocial) during the first half of the ERP paradigm. Increased brain activity related to reward processing indicated increased reward responsivity in adolescents with ASD, irrespective of stimulus type, after participating in a social skills intervention. A larger reward response is similar to what Kohls and colleagues [14] have described as a “liking” response involving the consumption of rewards that are salient. Initial sensitivity to rewards (e.g., during the first half of trials) may have been heightened after exposure to frequent reinforcement strategies that were utilized throughout the intervention to encourage participant engagement.

Although lack of significant differences in brain activity between groups at Time 1 (pre-intervention) is in contrast with some previous intervention literature utilizing neuroscience methods, e.g., [16], and changes in brain activity from pre- to post- intervention in individuals with ASD has been reported previously [17,18,20,21]. Notably, previous research measuring brain activity before and after intervention in individuals with ASD either did not utilize a neurotypical control group, e.g., [17,18,20,21], or had a neurotypical group but did not test children with ASD and the TD group at two timepoints (e.g., pre- and post-intervention for the ASD group). [16,22]. Collecting data from both teens with ASD and their neurotypical peers, as well as utilizing neuroscience paradigms that are hypothesized to capture changes directly relevant to the intervention itself, are both important strategies when measuring neural correlates of change after an intervention (for a review, see [39]). In the current study, we hypothesized that increased reward-related brain activity would be observed across the course of the ERP task after teens with ASD underwent an intervention that utilized social positive reinforcement principles to increase success in making and keeping friends. To our knowledge, this is the first investigation of brain activity of both neurotypical teens and those with ASD before and after participation in an intervention (or, in the case of the TD group, before and after a delay in which no intervention took place).

Contrary to our hypotheses, brain activity did not differ in response to condition (e.g., social, nonsocial) for either group. This contrasts with previous findings using this paradigm with young children with and without ASD [12,35]. However, this is the first time that this ERP paradigm has been utilized with adolescents. Thus, differences between the current study and previous research might reflect developmental changes. It is plausible that adolescents with and without ASD are less overtly motivated by food rewards as they would be by other reward types (e.g., monetary), and thus may have found the paradigm less engaging/rewarding than younger children. Future studies should consider utilizing this paradigm in a cross-sectional design with different age groups to better understand the effects of age on reward responsivity.

As expected, at Time 1 (pre-intervention), the ASD group had more severe social-communication impairments associated with ASD (measured by the SRS-2) and poorer social skills (measured by the SSIS social skills subscale) than the TD group. Adolescents with ASD improved on both measures after intervention (Time 2), which mirrors previously reported findings of the effectiveness of the PEERS^®^ social skills intervention [29,30]. No differences were observed from Time 1 to Time 2 in the TD group. This was expected, as the neurotypical teens did not participate in the intervention. Importantly, only one ASD participant remained in the range for clinical concern on both the overall SRS-2 score and SSIS social skills subscale score following intervention. This is important as it suggests that change from Time 1 to Time 2 was not only statistically significant, but also clinically meaningful. Further, no significant differences were observed between groups on the SRS-2 or SSIS social skills subscale at Time 2 (post-intervention), suggesting that both social-responsiveness symptoms and social skills in our sample of adolescents with ASD began to resemble social behaviors observed in our neurotypical participants.

One of the most interesting findings of our investigation was that ASD participants who demonstrated less robust social reward-related brain activity in the second half of trials prior to the intervention (Time 1) evidenced the biggest gains from Time 1 to Time 2 in both social responsivity and social skills. This suggests that perhaps the adolescents who benefitted the most from PEERS^®^ were those who had the most “room to improve” in terms of social reward response. This also provides initial evidence that the neural characteristics of reward responsiveness prior to intervention may serve as an indicator of treatment response. That is, it might be possible to utilize neural correlates of social reward responsivity to predict which individuals with ASD might benefit the most from participating in PEERS^®^. To further investigate this potential predictor of intervention efficacy, future research with a larger sample size and a randomized control group should be conducted.

## 6. Limitations

This study is part of a larger investigation of a social skills intervention, and this report serves as an initial analysis. Thus, the current study had a small number of participants. It is important to interpret differences in behavioral measures that were approaching significance with caution. Additionally, randomization of treatment was not performed (i.e., a waitlist control group was not utilized) and ASD participants were aware of their enrollment in the social skills intervention (i.e., parent rating forms were not completed “blind,” as parents were actively participating in the PEERS^®^ intervention with their teen). Thus, we cannot rule out the possibility that improvements in parent ratings in the ASD group were due to the expectation of improvements. Finally, findings from this study cannot be generalized to all individuals with ASD, as one of the criteria for participation was that the adolescent was motivated to participate in PEERS^®^ and wanted help making and keeping friends. Thus, this sample consisted of adolescents who were highly motivated to learn social skills.

## 7. Conclusions

The results of our study have important implications for intervention outcomes in adolescents with ASD. First, these findings add to the existing literature on the efficacy of PEERS^®^ for adolescents with ASD. Second, we found evidence for increased reward sensitivity in adolescents with ASD (compared to their neurotypical peers) after participation in the intervention. This suggests that participating in PEERS^®^ increases reward system sensitivity in teens with ASD. Finally, we found that teens who benefitted the most from the intervention (i.e., had the largest gains in social skills and largest decrease in social-communicative impairments) were those with less reward-related brain activity in response to faces prior to the intervention. This relationship between symptom improvement and brain activity prior to the intervention suggests that PEERS^®^ might be most effective for teens with ASD who have “room to grow” in their social reward responsivity, whereas teens with ASD who already have higher levels of social reward responsivity might benefit less. Finally, neuroscience measures may be reliable predictors of teens’ responsiveness to treatment because they are independent of potentially biased parent ratings.

## Figures and Tables

**Table 1:** Descriptive characteristics of the autism spectrum disorder (ASD) and neurotypical (TD) groups.

Variable	ASD	TD
Gender	6 male, 1 female	6 male, 1 female
Age in years, *M (SD), Range*	13.88 (2.21), 11.26–16.98	13.46 (2.29), 10.10–17.10
IQ, *M (SD), Range*	104.14 (17.36), 77–129	102.50 (17.96), 79–128
White *n*	2	1
Latino *n*	4	4
Mixed Race *n*	1	2
Maternal Education Level		
Less Than College	5	2
College and Above	2	3
*Missing Data*	0	2
Household Income		
Up to $50,000	3	1
$50,001–$100,000	2	1
Over $100,001	2	2
*Missing Data*	0	3

**Table 2:** Behavioral measures for Time 1 and Time 2 in ASD and TD groups.

Variable	ASD	TD
Time 1 *M (SD), Range*		
SRS-2	69.14 (14.18), 47–90	44.00 (4.55), 39–52
SSIS Social Skills	85.86 (25.13), 41–121	106.71 (11.93), 94–125
TASSK-R	14.29 (3.09), 10–9	14.57 (3.69), 10–21
Time 2 *M (SD), Range*		
SRS-2	61.43 (14.89), 45–88	48.00 (14.46), 39–80
SSIS Social Skills	93.57 (22.78), 51–120	105.00 (9.27), 96–119
TASSK-R	24.29 (4.61), 17–29	16.00 (2.65), 14–21
